# Tunable Metamaterial with Gold and Graphene Split-Ring Resonators and Plasmonically Induced Transparency

**DOI:** 10.3390/nano9010007

**Published:** 2018-12-21

**Authors:** Qichang Ma, Youwei Zhan, Weiyi Hong

**Affiliations:** Guangzhou Key Laboratory for Special Fiber Photonic Devices and Applications & Guangdong Provincial Key Laboratory of Nanophotonic Functional Materials and Devices, South China Normal University, Guangzhou 510006, China; qichangma@m.scnu.edu.cn (Q.M.); zhanyw@m.scnu.edu.cn (Y.Z.)

**Keywords:** metamaterials, mid infrared, graphene split-ring, gold split-ring, electromagnetically induced transparency effect

## Abstract

In this paper, we propose a metamaterial structure for realizing the electromagnetically induced transparency effect in the MIR region, which consists of a gold split-ring and a graphene split-ring. The simulated results indicate that a single tunable transparency window can be realized in the structure due to the hybridization between the two rings. The transparency window can be tuned individually by the coupling distance and/or the Fermi level of the graphene split-ring via electrostatic gating. These results could find significant applications in nanoscale light control and functional devices operating such as sensors and modulators.

## 1. Introduction

Electromagnetically-induced transparency (EIT) is a concept originally observed in atomic physics and arises due to quantum interference, resulting in a narrowband transparency window for light propagating through an originally opaque medium [1,2]. The EIT effect extended to classical optical systems using plasmonic metamaterials leads to new opportunities for many important applications such as slow light modulator [3,4,5,6], high sensitivity sensors [7,8], quantum information processors [9], and plasmonic switches [10,11,12]. Generally speaking, the realization of the EIT effect is usually achieved by two kinds of schemes such as the bright-bright mode coupling [7,12,13,14,15,16] and the bright-dark mode coupling [17,18,19,20,21]. So far, many researches have obtained the EIT effect from various metamaterial structures [11,22,23,24,25,26,27]. However, most of them only consist of metallic materials and they can only be controlled by the geometry of structures, which limits their applications.

Graphene is a two-dimensional material which is composed of single layer of carbon atoms, which has been widely used in optoelectronic devices, such as optical modulators [28,29,30] due to its unique properties such as high electron mobility [31], high light transparency [32] and high thermal conductivity [33]. Particularly, the surface conductivity of graphene has a wide range of tunable characteristics by changing the Fermi level of the graphene via electrostatic gating, which makes it a significant potential application in high-performance tunable optical devices [34,35]. Meanwhile, graphene is widely used in metamaterial to achieve a variety of optoelectronic devices with tunable properties [36,37,38].

Recently, L.S. Yan et al. have investigated that a plasmonic waveguide system based on side coupled complementary split-ring resonators and the electromagnetic responses of CSRR can be flexibly handled by changing the asymmetry degree of the structure and the width of the metallic baffles [39]. W. Yu et al. have realized a dual-band EIT effect which was composed of a split-ring resonator (SRR) and a pair of metal strips. Most of the previous researches were based on metallic split-ring which lacks tunability [40]. In this paper, we propose a periodic metamaterial structure which is composed of a graphene split-ring and a gold split-ring to investigate the EIT effect in the MIR regions. A transparency window can be observed in the transmission spectrum of the structure under plane wave excitation. It is found that the coupling strength greatly depends on the separation between the two rings. Besides, the EIT window can be tuned by changing the Fermi levels of the graphene split-ring via electrostatic gating, which may offer possible applications at tunable mid-infrared functional devices, such as optical switches and modulators.

## 2. Materials and Methods

**Figure 1:**
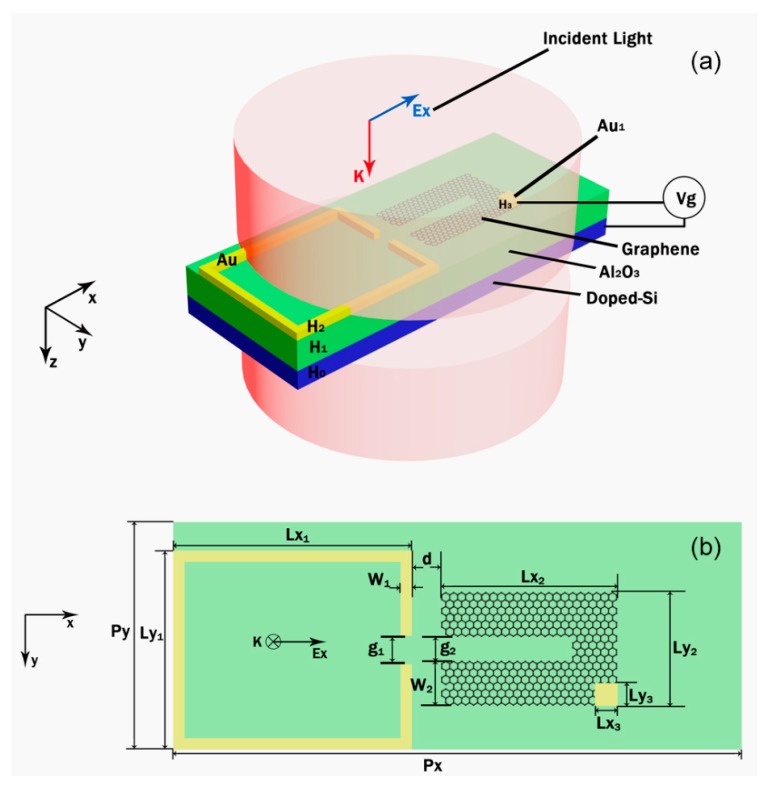
(**a**) Schematic diagram of the plasmonic metamaterial unit cell consists of a graphene split-ring and a gold split-ring. (**b**) Top view of metamaterial structure. The periods of the y and x direction are Py = 0.22 μm, Px = 1.34 μm, respectively. The distance between the graphene split-ring and the gold split-ring is *d* and the gaps of the gold split-ring and the graphene split-ring are g_1_ = 8 nm, g_2_ = 20 nm, respectively. The width of the gold split-ring and the graphene split-ring are W_1_ = 12 nm, W_2_ = 43 nm. The length of the gold split-ring in the y and x direction are Ly_1_ = 200 nm, Lx_1_ = 262 nm, respectively; and the length of the graphene split-ring in the y and x direction are Ly_2_ = 106 nm, Lx_2_ = 104 nm, respectively. The thickness of doped silicon, Al_2_O_3_ and Au are set as H_0_ = 10 nm, H_1_ = 40 nm and H_2_ = 4 nm, respectively. *V_g_* is the applied voltage and both the Au_1_ and doped silicon are served as electrodes to apply voltages to the graphene split-ring. The thickness of the Au_1_ is H_3_ = 4 nm and its width (Ly_3_) and length (Lx_3_) are set as 10 nm.

As shown in Figure 1a, the metamaterial structure used to demonstrate the EIT phenomenon consists of three layers. The top layer is composed of gold split-ring and graphene split ring while the middle layer plays a role as a substrate and its material is Al_2_O_3_ and the graphene split-ring is composed of 5 layers of graphene in our following simulations so as to enhance the modulation depth [41]. The bottom layer is doped silicon (doped Si) substrate which is used for an electrode to apply voltage to the graphene split-ring for changing its Fermi level, and another electrode is Au_1_ whose material is gold as shown in Figure 1a. In the simulation of the transmission spectra, Au_1_ is negligible because of the relatively small size with respect to the structure. The incident light propagates in the z direction and its polarization is along x direction. Both the gold split-ring and graphene split-ring are symmetric in y direction and their symmetry axis are coinciding. The (complex) refractive index of Al_2_O_3_ is obtained from Table I, page 662 in Ref. [42] and the doping concentration of doped Si is 10^18^cm^−3^ and its refractive index can be obtained from Ref. [43]. Both the Al_2_O_3_ and doped Si have been considered having the dispersion and absorption effects in our simulations.

The optical properties of the gold material are obtained from Palik’s experimental data. The surface complex conductivity of graphene *σ*(*ω*) is dominated by interband and intraband transitions within the random-phase approximation, which can be calculated as [44,45]:

(1) $$\sigma (\omega )=\sigma_{\mathrm{interband}}+\sigma_{\mathrm{intraband}}$$

where

(2) $$\sigma_{\mathrm{interband}}=\frac{e^{2}}{4\hbar }\{\frac{1}{2}+\frac{1}{\pi }\arctan\left(\frac{\hbar \omega -2E_{f}}{2k_{B}T}\right)-\frac{i}{2\pi }\ln\left(\frac{{\left(\hbar \omega +2E_{f}\right)}^{2}}{{\left(\hbar \omega -2E_{f}\right)}^{2}+{\left(2k_{B}T\right)}^{2}}\right)\}$$

(3) $$\sigma_{\mathrm{intraband}}=\frac{2ie^{2}k_{B}T}{\pi \hbar^{2}\left(\omega +i\tau^{-1}\right)}\ln\left(2\cosh\left(\frac{E_{f}}{2k_{B}T}\right)\right)$$

Here, *ω* is the optical angular frequency, *T* is the ambient temperature (300 K in this paper), *E_f_* is the Fermi level of graphene, *ħ* = *h*/2*π* is the reduced Plank’s constant, *k_B_* is the Boltzmann constant, *τ* is the carrier relaxation time and *τ* = *ħ*/2*Γ* [46], where *Γ* is the scattering rate and it is set 0.00099 eV in this paper (corresponding to the carrier relaxation time *τ* ≈ 0.33 ps). It can be seen from the above formula that the surface conductivity is greatly affected by the Fermi level and the Fermi level is determined by the carrier concentration *n_g_* and Fermi velocity *V_f_*. The formula *E_f_* = *ħV_f_(πn_g_)*^1/2^ with the Fermi velocity of *V_f_* = 10^6^ m/s can be obtained from Ref. [47] and *n_g_* = *ε*_0_*ε_d_V_g_*/*eH*_1_, where *V_g_* is the applied voltage and *ε_d_* is the permittivity of Al_2_O_3_ and H_1_ is the thickness of Al_2_O_3_. In the following simulation, graphene is modeled as a thin anisotropic material film and the in-plane permittivity is approximated to be [44]:

(4) $$\varepsilon_{g}=1+\frac{i\sigma (\omega )}{\varepsilon_{0}\omega t_{g}}$$

where *t_g_* is the thickness of graphene which is estimated as 1 nm [48] and *ε_0_* is the permittivity in vacuum. In addition, the conductivity of the N-layer graphene is N*σ* [49,50].

In this paper, we use the finite-difference time-domain (FDTD) simulations to numerically investigate the properties of the structure. The perfectly matched layer (PML) absorbing boundary conditions are set on the z direction while the periodic boundary conditions are set on the x and y directions.

## 3. Results and Discussion

**Figure 2:**
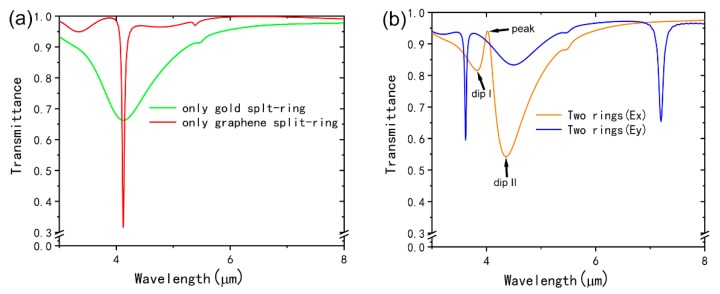
(**a**) Transmission spectra of the individual graphene split-ring and the individual gold split-ring. (**b**) Transmission spectra of the structure consists of graphene split-ring and gold split-ring under x-polarized and y-polarized incident light excitation, respectively.

To demonstrate the EIT effect, we numerically calculate the simulated transmission spectra of the unit cell with the graphene split-ring and the gold split-ring are shown in Figure 1a. The plasmonic resonances of the graphene split-ring and the gold split-ring are excited at the same wavelength. In addition, the transmission spectra of the unit cell with a graphene split-ring and a gold split-ring under x-polarized and y-polarized incident light excitation, respectively. It is obviously that when using the x-polarized incident light, a transparency window is observed. For the case of y-polarized incident light, there are two plasmon resonance peaks at 3.61 μm and 7.19 μm observed and no EIT phenomenon can be found. Consequently, we choose x-polarized incident light in the following simulations. In the simulations, the *E_f_* of the graphene split-ring is set as 0.9 eV and the coupling distance *d* is 20 nm. From the transmission spectra in Figure 2a, one can find that a strong plasmon resonance at 4.09 μm of both the graphene split-ring and the gold split-ring can be excited by the x-polarized incident light. The quality factor of graphene split-ring is higher than that of the gold split-ring and a transparency window with 95% transmission appears at 3.95 μm when we put the two rings together due to the interaction of them. In addition, two obvious dips can be found in the transmission spectra (orange line in Figure 2b) at 3.83 μm and 4.35 μm, respectively.

**Figure 3:**
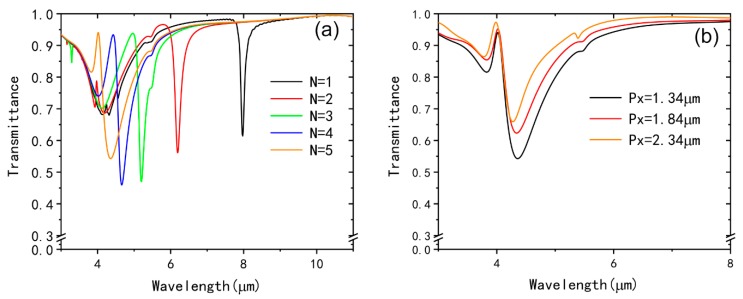
(**a**) Transmission spectra of the individual graphene split-ring with different layers. (**b**) Transmission spectra of the structure with different periods (Px) in the x direction.

Then, we study the response of EIT effect resulting from the variation of the layers of graphene split-ring (Figure 3a) and the periods in the x direction the total structure. The Fermi level is set as 0.9 eV and the distance of the two split-rings *d* is 20 nm. It can be observed that dip II is obviously blue-shifted from 7.97 μm to 4.35 μm while dip I blue-shifts slightly and its transmittance increases with the layers of the graphene split-ring N increasing from 1 to 5. In addition, it is clear shown that the transmission peak is blue-shifted and the line width of the transmission peak becomes smaller and narrower. This is because the hybridized of the gold split-ring and graphene split-ring become stronger with increasing the layer number N. It is also found that 4 layers or 5 layers of the graphene split-ring are suitable. Here we choose the graphene split-ring of 5 layers because the quality factor of transmission peak is larger than that of the 4 layers. In Figure 3b, it is shown that the period in the x direction of the plasmonic metamaterial unit cell can also influence the EIT phenomenon. With the increase of the Px from 1.34 μm to 2.34 μm, both dip I and dip II blue-shift slightly and the amplitudes of the transmittance increase. The line width of the transmission peak becomes larger and its transmittance increase with increasing the periods (Px) in the x direction.

**Figure 4:**
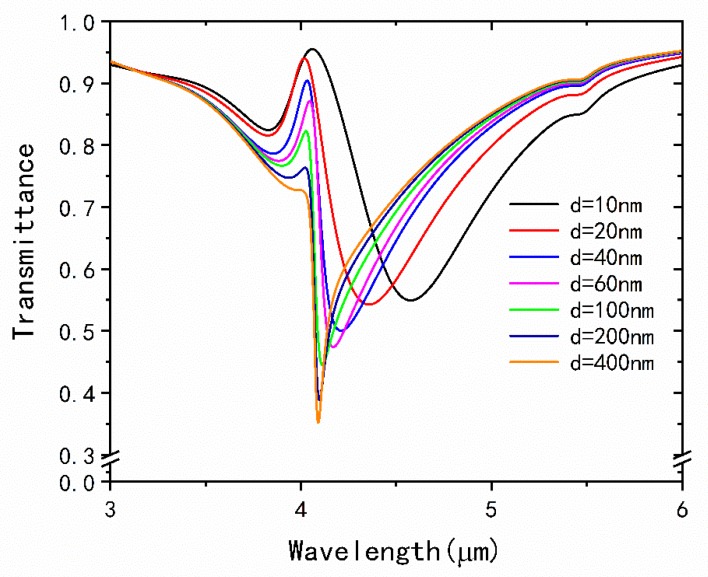
Transmission spectra of the metamaterial structure with different distances *d*.

Furthermore, we also investigate the dependence of the EIT spectra on the distance *d* between the two rings. As shown in Figure 4, the EIT peak gradually becomes sharper and its transmittance gradually decreases with the increasing of *d*. When *d* comes to 400 nm, the peak will disappear. It reveals that the interaction of gold split-ring and graphene split-ring becomes weaker and their interaction eventually disappears when the coupling distance is large enough. It can also find that dip I red-shifts slightly while the dip II blue-shifts with the increase of *d* and both their transmittance decrease. Therefore, the coupling strength of the metamaterial structure greatly depends on the distance of the gold split-ring and graphene split-ring.

**Figure 5:**
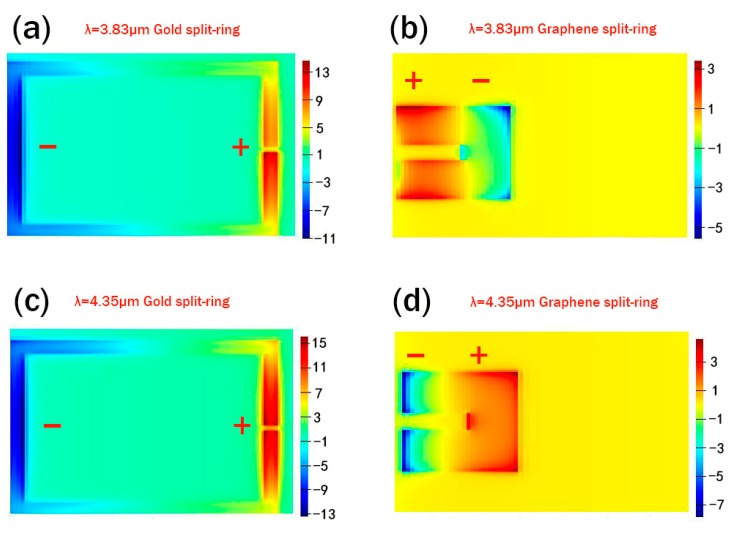
z-component distributions of the electric field under x-polarized continue light excitation at (**a**) *λ* = 3.83 μm (Au power monitor), (**b**) *λ* = 3.83 μm (graphene power monitor), (**c**) *λ* = 4.35 μm (Au power monitor), (**d**) *λ* = 4.35 μm (graphene power monitor).

To get further insight into the physics of the EIT phenomenon of our structure, the electric-field distributions of the z-component at wavelength I (*λ* = 3.83 μm) and wavelength II (λ = 4.35 μm) are shown in Figure 5a–d. The generation of dip I and dip II can be understood and predicted by the plasmon hybridization model, similar to the case for the molecular orbital theory and plasmon hybridization model [51,52,53,54]. Since the thicknesses of the graphene split-ring and the gold split-ring are different, we place two power monitors at 2 nm above the graphene split-ring and the gold split-ring, respectively. In this simulation, the distance *d* is 20 nm, and the Fermi level of graphene split-ring is 0.9 eV.

The placement of graphene split-ring and gold split-ring can be treated as asymmetric nanoparticle dimer which has been investigated in Ref. [53]. When *d* is small enough, their local surface plasmons will couple to each other, which is analogous to σ hybridization of *p* orbital electron in the atomic outer layer. From Figure 5a,b, one can observe that the left side of the gold split-ring gathers a large amount of negative induced charge, and a large amount of positive induced charges are accumulated on the right side for the case of *λ* = 3.83 μm. In contrast, the positive and negative induced charges are distributed on the left and right sides of the graphene split-ring. The different charge distributions of the two rings are similar to the anti-bonding mode of the hybrid molecular system. On the other hand, one can find that the distribution of induced change in the gold split-ring remains unchanged at the wavelength *λ* = 4.35 μm, while the distribution of induced change in the graphene split-ring is opposite to the situation at *λ* = 3.83 μm from Figure 5c,d, which is similar to the bonding mode of hybrid molecular system.

**Figure 6:**
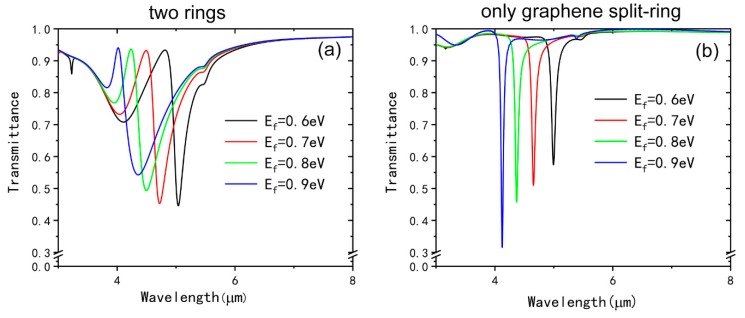
(**a**) Transmission spectra of the two rings with different Fermi levels of the graphene split-ring. (**b**) Transmission spectra of the individual graphene split-ring with different Fermi levels.

As mentioned earlier, the complex surface conductivity of graphene can be changed by controlling its Fermi level, which can be realized by applying different gate-voltages to the graphene split-ring. Compared with the reported bright-bright mode coupling EIT structure fabricated by metallic material, we can realize the dynamically control of the EIT window in our metamaterial structure without reconstructing the geometries or imbedding other actively controlled materials. Figure 6a illustrates the transmission spectra with different Fermi levels, it is apparent that the EIT window can be tuned over a large range in the MIR regions by a small change of the Fermi level. When the Fermi level changes from 0.6 eV to 0.9 eV, the transmission window blue-shifts from 4.82 μm to 4.02 μm, which can be interpreted by the graphene plasmonic resonance wavelength *λ~1/E_f_^1/2^* [55]. At the same time, the transmittance of the dip I and dip II will decrease with increasing of the Fermi level while the transmittance of the peak almost remains unchanged. The transmission spectra of the unit cell with only graphene split-ring with different Fermi level are shown in Figure 6b. When the Fermi level changes from 0.6 eV to 0.9 eV, the resonance wavelengths blue shift from 4.99 μm to 4.12 μm and the amplitude of the transmittance are decreasing. It is easy to find that the resonance wavelength of individual graphene split-ring in Figure 6a is almost the same as Dip II in Figure 6b for the same Fermi level, which shows that the dip II is greatly affected by the graphene split-ring.

## 4. Conclusions

In this paper, we have successfully proposed that the EIT effect can be realized through bright-bright modes coupling in the MIR regions. The EIT window appears due to the hybridization between the gold split-ring and graphene split-ring and it is sensitive to the coupling distance. Moreover, we investigate the response of EIT effect resulting from the variation of the layers of graphene split-ring and periods in the x direction. Particularly, the EIT window can be dynamically tuned by adjusting the Fermi level of the graphene split-ring instead of reconstructing the structure. These properties could lead to new opportunities for many important applications, such as sensors and slow light modulators in MIR regions.
